# Deep learning model for genotype prediction from echocardiographic videos in non-ischaemic dilated cardiomyopathy

**DOI:** 10.1093/ehjdh/ztag068

**Published:** 2026-05-05

**Authors:** Yuko Kiyohara, Seito Fukagawa, Seitaro Nomura, Satoshi Kodera, Koki Nakanishi, Takashi Hiruma, Ryo Abe, Shunsuke Inoue, Junichi Ishida, Eisuke Amiya, Masaru Hatano, Hiroyuki Morita, Norihiko Takeda, Issei Komuro

**Affiliations:** Department of Frontier Cardiovascular Science, Graduate School of Medicine, The University of Tokyo, 7-3-1 Hongo, Bunkyo-ku, Tokyo 113-8655, Japan; Department of Frontier Cardiovascular Science, Graduate School of Medicine, The University of Tokyo, 7-3-1 Hongo, Bunkyo-ku, Tokyo 113-8655, Japan; Department of Frontier Cardiovascular Science, Graduate School of Medicine, The University of Tokyo, 7-3-1 Hongo, Bunkyo-ku, Tokyo 113-8655, Japan; Department of Cardiovascular Medicine, Graduate School of Medicine, University of Tokyo, 7-3-1 Hongo, Bunkyo-ku,Tokyo 113-8655, Japan; Department of Cardiovascular Medicine, Graduate School of Medicine, University of Tokyo, 7-3-1 Hongo, Bunkyo-ku,Tokyo 113-8655, Japan; Department of Cardiovascular Medicine, Graduate School of Medicine, University of Tokyo, 7-3-1 Hongo, Bunkyo-ku,Tokyo 113-8655, Japan; Department of Frontier Cardiovascular Science, Graduate School of Medicine, The University of Tokyo, 7-3-1 Hongo, Bunkyo-ku, Tokyo 113-8655, Japan; Department of Cardiovascular Medicine, Graduate School of Medicine, University of Tokyo, 7-3-1 Hongo, Bunkyo-ku,Tokyo 113-8655, Japan; Department of Frontier Cardiovascular Science, Graduate School of Medicine, The University of Tokyo, 7-3-1 Hongo, Bunkyo-ku, Tokyo 113-8655, Japan; Department of Cardiovascular Medicine, Graduate School of Medicine, University of Tokyo, 7-3-1 Hongo, Bunkyo-ku,Tokyo 113-8655, Japan; Department of Frontier Cardiovascular Science, Graduate School of Medicine, The University of Tokyo, 7-3-1 Hongo, Bunkyo-ku, Tokyo 113-8655, Japan; Department of Cardiovascular Medicine, Graduate School of Medicine, University of Tokyo, 7-3-1 Hongo, Bunkyo-ku,Tokyo 113-8655, Japan; Department of Cardiovascular Medicine, Graduate School of Medicine, University of Tokyo, 7-3-1 Hongo, Bunkyo-ku,Tokyo 113-8655, Japan; Department of Cardiovascular Medicine, Graduate School of Medicine, University of Tokyo, 7-3-1 Hongo, Bunkyo-ku,Tokyo 113-8655, Japan; Department of Cardiovascular Medicine, Graduate School of Medicine, University of Tokyo, 7-3-1 Hongo, Bunkyo-ku,Tokyo 113-8655, Japan; Department of Cardiovascular Medicine, Graduate School of Medicine, University of Tokyo, 7-3-1 Hongo, Bunkyo-ku,Tokyo 113-8655, Japan; International University of Health and Welfare, 4-1-26 Akasaka, Minato City, Tokyo 107-8402, Japan; Department of Cardiovascular Medicine, Graduate School of Medicine, University of Tokyo, 7-3-1 Hongo, Bunkyo-ku,Tokyo 113-8655, Japan; Department of Frontier Cardiovascular Science, Graduate School of Medicine, The University of Tokyo, 7-3-1 Hongo, Bunkyo-ku, Tokyo 113-8655, Japan; International University of Health and Welfare, 4-1-26 Akasaka, Minato City, Tokyo 107-8402, Japan

**Keywords:** DCM, Genotype, Deep learning, Echocardiography

## Abstract

**Aims:**

Non-ischaemic dilated cardiomyopathy (DCM) is frequently characterized by the presence of pathogenic germline variants, and genotype positivity predicts poor prognosis. Despite its importance, genetic testing remains underutilized in the current era. Therefore, we aimed to develop a deep learning model to predict genotype positivity using echocardiographic videos.

**Methods and results:**

We included patients who were diagnosed with DCM and had genetic testing at the University of Tokyo Hospital, Japan, consecutively from 2014 to 2022. The apical four-chamber views of echocardiographic videos were collected. First, we developed a deep learning model based on the EchoNet-Dynamic model, and the area under the curve (AUC) was computed. Second, we calculated the Madrid genotype score (clinical scoring system) for each case. Third, we developed a logistic regression model that combined the Madrid genotype score and the deep learning model. Finally, we compared the AUC of the combined model with that of the Madrid genotype score alone by DeLong’s test. Out of the 258 patients, 117 patients (45.3%) had genetic variants, and 141 (54.7%) did not. *TTN* (30.8%) was the most common genotype, followed by *LMNA* (18.8%). The deep learning model yielded an AUC of 0.64. The Madrid genotype score was well validated and achieved an AUC of 0.73. The combined model yielded an AUC of 0.76 with a significant improvement from the Madrid genotype score alone (*P* = 0.03).

**Conclusion:**

The deep learning model demonstrated modest discriminative ability to predict genotype positivity using echocardiographic videos. The accuracy of the clinical scoring system improved when combined with the deep learning model.

## Graphical Abstract

**Graphical Abstract:**
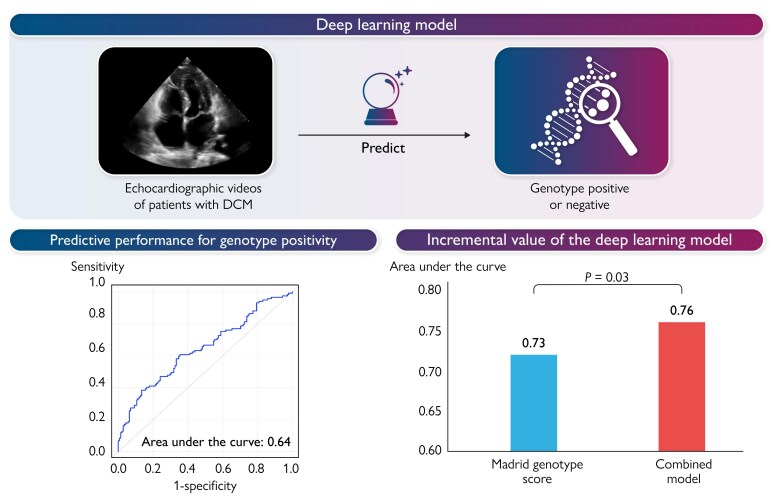
DCM, dilated cardiomyopathy.

## Introduction

Non-ischaemic dilated cardiomyopathy (DCM) is a significant cause of heart failure, affecting ∼1 in 2500 individuals and sometimes necessitates heart transplantation due to its refractory nature to medical treatment.^1^ To date, more than 60 genes have been categorized as linked to DCM in the Human Gene Mutation Database.^2^ These genes encode proteins within the cardiomyocyte, including sarcomere components, cytoskeletal proteins, and nuclear envelope proteins.^3^ Recent studies showed that these gene variants were found in up to 40% of patients with DCM.^1,4,5^ Furthermore, patients who carry these genetic variants tend to have worse clinical outcomes than those who do not.^4,5^ In particular, DCM with lamin A/C gene (*LMNA*) mutation is associated with a high incidence of cardiac events, including ventricular arrhythmia.^6^

The identification of causative variants enables the stratification of patients with DCM into subpopulations that benefit from further treatments. The current guideline recommends genetic testing for all patients with DCM, regardless of age, and sets a lower threshold of implantable cardioverter-defibrillator (ICD) for primary prevention of sudden cardiac death in patients with selected genetic variants (Class IIa/IIb).^1^ Despite this recommendation, genetic testing remains underutilized in clinical practice. One previous report from the USA showed that only 0.8% of patients newly diagnosed with DCM underwent genetic testing owing to human and financial constraints.^7^ To address this issue, prioritization for genetic testing based on predicted genotype positivity from clinical information is warranted.

Recently, deep learning has developed and is widely applied in the field of medicine, including cardiology.^8^ One of the applications is echocardiography, which is widely available and provides detailed information on cardiac structure and function. The EchoNet-Dynamic model was reported to precisely predict left ventricular ejection fraction (LVEF).^9^ Based on this model, several models were developed to predict some diseases, including occult atrial fibrillation and cardiac sarcoidosis.^10,11^ However, no previous reports investigated the echocardiographic videos in patients with DCM to predict genotype positivity based on echocardiographic findings. Herein, we aimed to develop a deep learning model to predict genotype positivity using echocardiographic videos of patients with DCM.

## Methods

### Overall

We included DCM patients with echocardiograms stored in the medical health record. The four-chamber views of echocardiographic videos were selected. After labelling these videos as genotype positive or genotype negative, we developed a model based on the EchoNet-Dynamic model, which used a convolutional neural network (CNN) and was already trained by the EchoNet-Dynamic dataset.^9^ We applied five-fold cross-validation. Finally, we calculated the area under the curve (AUC). The study was approved in accordance with the ethics committee at the University of Tokyo Hospital (approval number: G2249) and conducted in accordance with the principles of the Declaration of Helsinki.

### Population

We consecutively included patients who were diagnosed with DCM and had genetic testing at the University of Tokyo Hospital from 2014 to 2022. The diagnosis of DCM was made clinically by well-experienced cardiologists based on the conventional definition after excluding ischaemic aetiology.^12,13^ Ischaemic aetiology was excluded in accordance with current clinical guidelines, based on findings from electrocardiography, transthoracic echocardiography, and coronary angiography performed when clinically indicated.

The exclusion criteria were as follows: (i) those with no echocardiogram data restored in the medical health record and (ii) those with no echocardiogram data before left ventricular assist device (LVAD) implantation or heart transplantation. The four-chamber views of echocardiographic videos were selected in patients without LVAD or heart transplantation. Also, we collected patient characteristics and key parameters at the initial echocardiogram from electronic medical records.

### Genetic testing for germline variants in genes related to dilated cardiomyopathy

Whole blood samples were collected from participants, and DNA was extracted from the samples. We performed targeted sequencing or whole-exome sequencing for the prespecified set of genes harbouring variants that are known to alter protein structures or function in cardiomyopathy (see Supplementary material online, *Table S1*), and additional details of this process were published elsewhere.^14^ Detected variants were classified into pathogenic, likely pathogenic, benign, likely benign, and uncertain significance according to the American College of Medical Genetics and Genomics guidelines.^15^ Variants classified as pathogenic or likely pathogenic were considered pathogenic variants in the current study.

### Madrid genotype score

We calculated the Madrid genotype score in this cohort to evaluate the efficacy of a recently reported clinical scoring for patients with DCM.^16^ This scoring system was the first one to predict the probability of a positive genetic test result in DCM. This score was based on the following five clinical features: skeletal myopathy, family history of DCM, low voltage on electrocardiogram, absence of hypertension, and absence of left bundle branch block. We collected the above information through chart review and calculated the score for each patient.

### Dataset and labelling

The echocardiographic videos were acquired by experienced sonographers or cardiologists at the University of Tokyo Hospital. The ultrasound machines included the following four: Vivid 7/Vivid E9/Vivid E95 (GE Healthcare, Waukesha, Wisconsin, USA), iE33/EPIQ7 (Philips, Amsterdam, the Netherlands), Xario/Artida/Aplio300/AplioXV (Toshiba, Tokyo, Japan), and Acuson SC2000 (Siemens, Munich, Germany). Out of the echocardiographic videos of the included patients, apical four-chamber views were collected in the current study. To remove personality identifiable information originally in the videos, we cropped them and omitted text, electrocardiogram, and other information outside of the scanning sector. Following the crop, the square movies had 370 × 370, 380 × 380, 470 × 470, 550 × 550, 600 × 600, or 630 × 630 pixels, depending on the machine. Using cubic interpolation, these cropped videos were down-sampled into standardized 112 × 112 pixels and saved as Audio Video Interleave files. The training dataset was augmented using colour inversion, Gaussian blur, and horizontal flipping. We labelled these videos as genotype positive or negative.

### Deep learning model

Our model was based on the EchoNet model, which used a video-based deep learning algorithm for predicting LVEF.^9^ The head of our model was a classifier made by CNN. Our model had spatiotemporal convolutions, which allow the simultaneous analysis of the image in each frame and its change over time. We used transfer learning because of the insufficient amount of data for learning neural network models. First, the parameters of our model were set after pre-training using the EchoNet-Dynamic dataset.^9^ Then, the parameters were updated using our training dataset. The entire process was summarized in Supplementary material online, *Figure S1*.

### Training and test

We first separated each dataset into training, validation, and test datasets and applied a five-fold cross-validation to precisely evaluate our model. Each data was randomly divided into five subsets in a way that each subset might have the same ratio of genotype positive and genotype negative. Out of the five subsets, three were used as training datasets, one as a validation dataset, and the remaining one as a test dataset to evaluate our classification model. The process is repeated multiple times, with each subset taking turns being the validation and test datasets.

After pre-training with the EchoNet-Dynamic dataset, the model was trained using the training dataset and their augmented versions. For each echocardiographic video, three separate movie clips of 32 frames were generated as input data. To classify genotype positivity, our algorithms were trained to minimize the binary cross-entropy loss between predictions and ground truth using the Adam optimizer, a widely adopted variant of stochastic gradient descent, with an initial learning rate of 0.00001, a batch size of eight, and for 100 epochs. The learning rate was reduced if the validation loss plateaued after four epochs. If the validation loss did not improve for 10 consecutive epochs, model training was stopped before 100 epochs were completed, and the model’s weights corresponding to the lowest validation loss were saved.

The developed algorithms were applied to the test dataset to evaluate their diagnostic performance. However, because the input video comprised 32 consecutive frames randomly cut from one video, different inputs could be made to the algorithm for each evaluation. Therefore, the evaluation was performed 10 times, and the average value of each evaluation was used as the predicted value. The whole process of model designing, training, and testing was done in Python using the PyTorch deep learning library.^17^ Also, we use a Nvidia Tesla V-100 32 GB graphics processing unit.

### Performance evaluation

To evaluate the performance of the prediction for genotype positivity, we calculated AUC. When multiple echocardiographic videos existed for a single case, the average of the predicted values from each video was used as the predicted value for that case. We compared echocardiographic features between cases predicted as genotype positive and those predicted as genotype negative by the deep learning model.

### The combination of the deep learning model with the Madrid genotype score

We calculated the predicted probability based on the Madrid genotype score for each case of our cohort and calculated an AUC.^16^ Then, we developed a logistic regression model, trained with cross-validation, to generate a new prediction by combining our deep learning model’s predicted value and the Madrid genotype score. Subsequently, we calculated an AUC of the combined model. DeLong’s test was applied to compare these two AUCs to investigate the incremental value of the deep learning model. A *P* value of <0.05 was considered statistically significant.

### Deep learning models to predict *LMNA* and sarcomere variants

We developed additional models to predict specific subtypes of genotypes using the same methodology as the main analysis. First, we conducted a deep learning model to predict *LMNA* pathogenic variants and calculated its discriminative performance. Second, we conducted a deep learning model to predict sarcomere gene variants, including *TTN*, *MYBPC3*, and *MYH7*, and calculated its AUC.

## Results

### Patient characteristics and echocardiographic parameters at baseline

**Figure 1:**
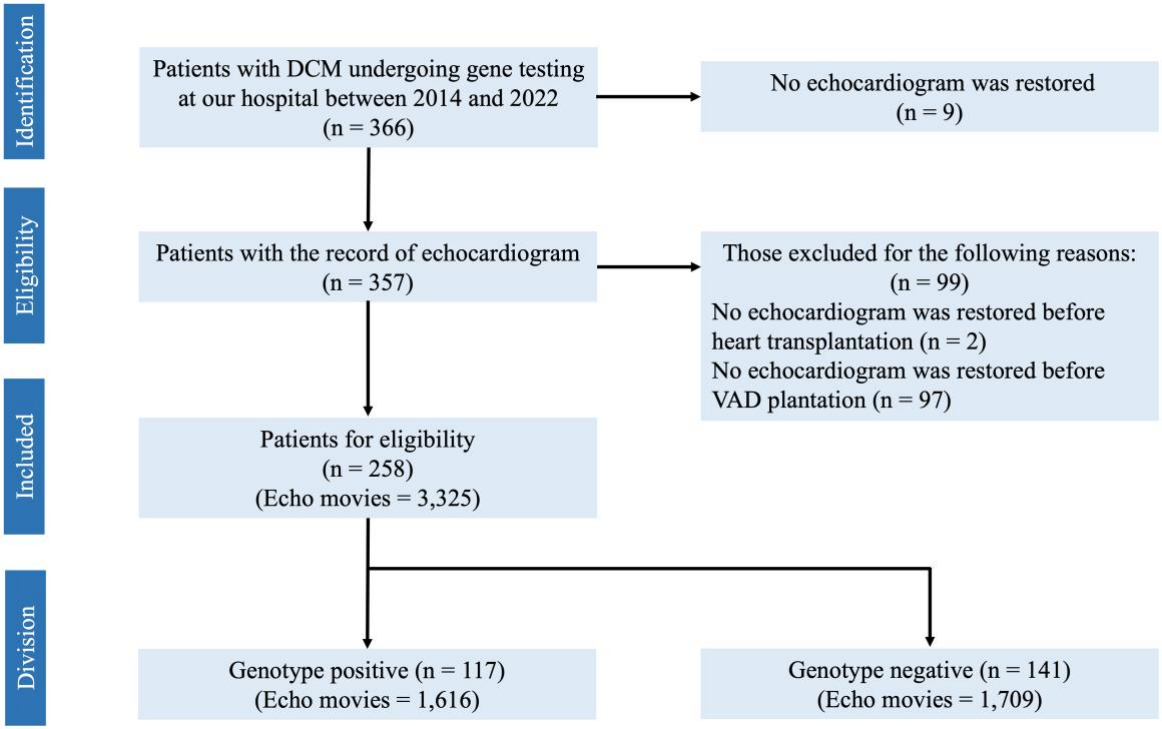
Flow chart of participant selection. DCM, dilated cardiomyopathy; VAD, ventricular assist device.

Among 258 patients with DCM, 117 patients carried pathogenic variants (genotype-positive group), and 141 had no pathogenic variants (genotype-negative group) (*Figure 1*). We collected 3325 four-chamber apical views of echocardiographic videos. The genotype-positive group was associated with younger age and smaller left ventricular (LV) end-diastolic diameter, compared with the genotype-negative group (*Table 1*). There was no significant difference in LVEF between the two groups. Among 117 patients with pathogenic variants, *TTN* was the most common (30.8%), followed by *LMNA* (18.8%) (*Table 2*). The detailed summary of variants identified in cardiomyopathy-related genes is shown in Supplementary material online, *Table S2*.

**Table 1:** Baseline characteristics

	Genotype positive (*n* = 117)	Genotype negative (*n* = 141)	*P* value
Clinical characteristics			
Age (years)	42 ± 14	48 ± 13	0.001
Male	85/117 (73%)	99/141 (70%)	0.770
Height (cm)	167 ± 9	168 ± 9	0.289
Weight (kg)	63 ± 16	67 ± 16	0.077
Smoking	65/112 (58%)	72/135 (53%)	0.541
Hypertension	10/113 (9%)	30/137 (22%)	0.009
Diabetes mellitus	16/114 (14%)	27/137 (20%)	0.308
Dyslipidaemia	17/115 (15%)	40/137 (29%)	0.010
Chronic kidney disease	9/109 (8%)	23/133 (17%)	0.061
Echocardiographic characteristics			
LV end-diastolic diameter (mm)	64 ± 11	67 ± 12	0.035
LV end-systolic diameter (mm)	57 ± 13	59 ± 13	0.078
LVEF (%)	27 ± 14	27 ± 13	0.784
RVSP (mmHg)	32 ± 14	32 ± 13	0.750
TAPSE (mm)	17 ± 5	17 ± 7	0.624

The patient characteristics at the time of the first echocardiogram in our hospital are shown. Data are *n*/*N* (%) or the mean ± standard deviation. *P* values are from Fisher’s exact test for categorical variables, and the Kruskal–Wallis test for continuous variables

LV, left ventricle; LVEF, left ventricular ejection fraction; RVSP, right ventricular systolic pressure; TAPSE, tricuspid annular plane systolic excursion.

**Table 2:** List and proportion of the genes

Gene	Number of cases	Proportion of cases (%)
*TTN* ^a^	35	29.9
*LMNA* ^b^	20	17.1
*TNNT2*	14	12.0
*FLNC*	8	6.8
*BAG3*	6	5.1
*DSP*	6	5.1
*DES*	5	4.3
*MYH7*	4	3.4
*RBM20*	3	2.6
*DSG2*	2	1.7
*EMD*	2	1.7
*SCN5A*	2	1.7
*TNNC1*	2	1.7
*TPM1*	2	1.7
*ACTC1*	1	0.9
*ACTN2*	1	0.9
*LMNA* ^b^ *, MYH7*	1	0.9
*LMNA* ^b^ *, TTN* ^a^	1	0.9
*NEXN*	1	0.9
*TNNI3*	1	0.9

A total of 117 patients (45.3%) had at least one genetic variant. Among the genotype-positive patients, *TTN* was the most common (30.8%), followed by *LMNA* (18.8%).

^a^ *TTN* was detected in 30.8%, including one case: [*LMNA*, *TTN*].

^b^ *LMNA* was detected in 18.8%, including two cases: [*LMNA*, *MYH7*] and [*LMNA*, *TTN*].

### The performance of the deep learning model, the Madrid genotype score, and the combined model

**Figure 2:**
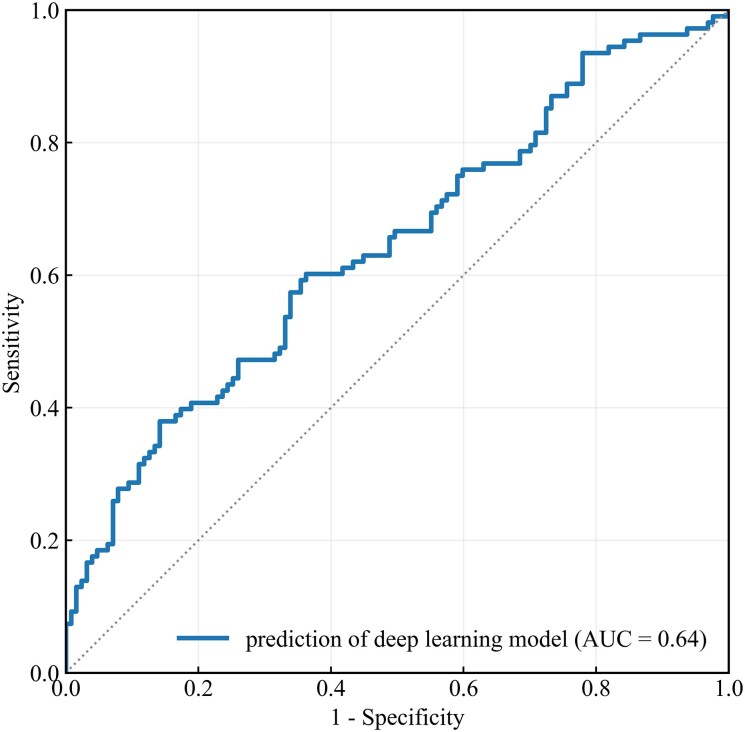
The ROC curve of the deep learning model in predicting genotype positivity. The AUC of the model was calculated as 0.64 (95% CI 0.58–0.71). AUC, area under the curve; CI, confidence interval; ROC, receiver operating characteristic.

**Figure 3:**
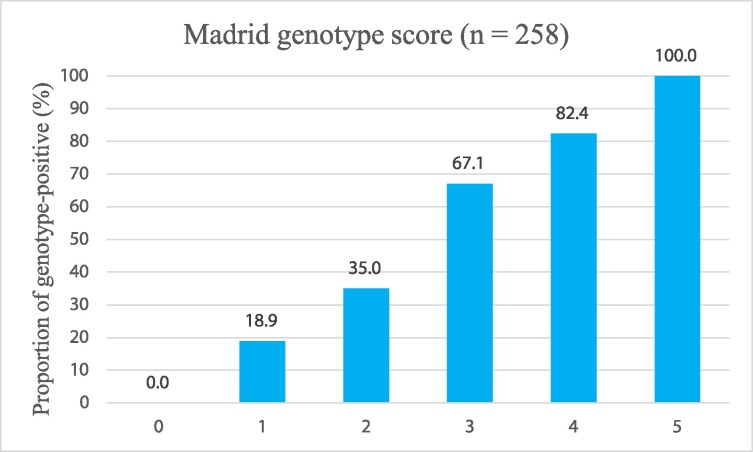
The yield of genotype-positive results according to the Madrid genotype score. The proportion of genotype-positive cases was higher with a higher Madrid genotype score.

**Figure 4:**
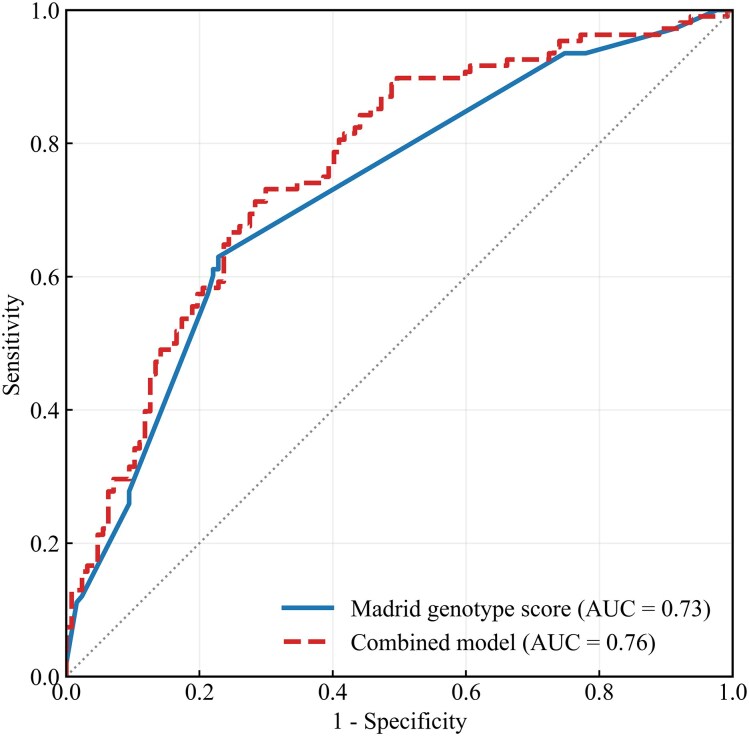
The ROC curves of the Madrid genotype score and the combined model. The ROC curves of the Madrid genotype score and the combined model are shown. The Madrid genotype score had an AUC of 0.73 (95% CI 0.67–0.79). The combined model had an AUC of 0.76 (95% CI 0.70–0.82). AUC, area under the curve; CI, confidence interval; ROC, receiver operating characteristic.

The deep learning model had an AUC of 0.64 [95% confidence interval (CI) 0.58–0.71]. (*Figure 2*). Regarding echocardiographic features, LV end-diastolic and end-systolic diameters were significantly different between cases predicted as genotype positive and those predicted as genotype negative by the deep learning model (see Supplementary material online, *Table S3*). The proportion of genotype-positive cases increased with higher scores of the Madrid genotype score (*Table 3*). The probability of genotype-positive results in this cohort ranged from 0% with zero points, 82.4% with four points, and 100% with five points (*Figure 3*). Madrid genotype score alone achieved an AUC of 0.73 (95% CI 0.67–0.79), and the combined model yielded an AUC of 0.76 (95% CI 0.70–0.82) (*Figure 4*). DeLong’s test demonstrated a statistically significant difference between the combined model and the Madrid genotype score (*P* = 0.03).

**Table 3:** Genetic yield for each of the Madrid genotype scores

Madrid genotype score	Genotype positive	Genotype negative	Total
	(*n* = 117)	(*n* = 141)	(*n* = 258)
0	0	4 (3)	4 (2)
1	7 (6)	30 (21)	37 (14)
2	42 (36)	78 (55)	120 (47)
3	53 (45)	26 (18)	79 (31)
4	14 (12)	3 (2)	17 (7)
5	1 (1)	0	1 (0.4)

The *n* (%) of cases categorized into each of the Madrid genotype scores are shown.

### Deep learning models to predict *LMNA* and sarcomere variants

The deep learning model for predicting *LMNA* pathogenic variants achieved an AUC of 0.49, whereas the model for sarcomere gene variants achieved an AUC of 0.59 (see Supplementary material online, *Figure S2*).

## Discussion

In the current study, we developed a deep learning model to predict genotype positivity using echocardiographic videos and combined the deep learning model with the Madrid genotype score. The results of our study can be summarized as follows: (i) the deep learning model predicted the existence of pathogenic variants at moderate accuracy, (ii) the Madrid genotype score was well validated in the Japanese cohort, and (iii) the accuracy of the Madrid genotype score was enhanced when combined with the deep learning model. To the best of our knowledge, our model is the first one to predict genotype positivity based on echocardiographic findings of patients with DCM.

Our study, conducted in an exclusively Asian cohort, revealed that ∼40% of the included population had pathogenic variants, and the Madrid genotype score was validated in this cohort. This proportion was concordant with previous reports from Western countries, with 37% in Spain and the USA^5^ and 37% in Italy.^4,5^ In the reports, *TTN* was the most frequent, followed by *LMNA*, which was consistent with our result. In terms of racial disparity of DCM, although the incidence is reportedly higher in Black patients than in White patients, the prevalence in the Asian population remains unclear.^18,19^ The Madrid genotype score was developed using Spanish, Dutch, and Italian cohorts.^16^ Our study showed that this clinical scoring system was validated in our Japanese cohort, suggesting the clinical validity of this scoring system across races and countries.

Our deep learning model yielded moderate accuracy in predicting genotype positivity in patients with DCM using echocardiographic videos. The interpretability of the model remains limited since the specific echocardiographic features driving genotype discrimination have not been fully identified. Importantly, LVEF was not significantly different between the two groups, although our model was developed based on the EchoNet-Dynamic model, which was developed to predict LVEF. Our findings suggested that the deep learning model might, at least in part, be associated with features related to LV end-diastolic and end-systolic diameters. Importantly, however, these parameters alone do not fully explain the model’s predictions, as they show substantial overlap between groups and are insufficient to account for the genotype discrimination on their own. At present, conventional echocardiographic differences between genotype-positive and genotype-negative groups remain incompletely understood, making it difficult to define clear reference features based solely on parameters that can currently be quantified and evaluated. Nevertheless, our findings support the possibility that the model might have detected more subtle spatiotemporal patterns of LV remodeling across multiple echocardiographic views, rather than relying on a single static parameter. Notably, combined with the clinical scoring system, the deep learning model achieved higher accuracy. These findings suggest that the integration of clinical information with echocardiographic videos may represent a promising strategy for predicting genotype positivity in future clinical practice.

Echocardiographic videos have the potential to be used as a screening tool to predict genotype positivity in the current situation of the underutilization of genetic testing. One recent report based on the claims data in the USA showed that only 1.1% of patients with cardiomyopathy underwent genetic testing.^20^ The current low rate of genetic testing is primarily due to the limited availability of necessary equipment and inadequate reimbursement policies, highlighting substantial room for improvement.^21^ On the other hand, echocardiography is widely available in daily practice for patients with DCM, as the current guideline recommends echocardiography for all cases of DCM.^22^ Given the limited availability of genetic testing, echocardiography could serve as a screening tool for predicting the existence of pathogenic variants through the enhancement of model performance using a large volume of echocardiographic videos for DCM.

Our study has some similarities to the recent concept of radiogenomics, addressing the relationship between imaging phenotypes and genomic information.^23^ This concept has been regarded as a key concept for precision medicine, especially in oncology.^24^ Magnetic resonance imaging is commonly used as the modality for radiogenomics.^23,24^ Echocardiographic videos can have the potential for this concept, and we can progress towards precision medicine by training deep learning models using echocardiographic videos to detect genetic information.

There are some limitations in our study. First, due to the small number of cases, overfitting could not be completely avoided. The number of our cases is relatively small because genetic testing is not commonly performed. Although we tried our best to prevent overfitting by performing data augmentation and using cross-validation, there is still a possibility of overfitting. The exploratory models to predict subtype variants had relatively low discriminative performance, possibly due to the limited number of cases. A larger sample size would help us avoid overfitting; thus, the accuracy is expected to improve. Second, the evaluation of our model is difficult since there are no established criteria to diagnose DCM based on genomic information or on echocardiographic findings. That is, it is difficult to evaluate whether our results had sufficient accuracy or not due to the scarcity of existing findings. Third, potential confounders related to clinical parameters were not fully adjusted for. Lastly, the detected variants in this study are not necessarily comprehensive. In the current study, genotype-positive cases were defined based on pathogenic or likely pathogenic variants identified in known cardiomyopathy-associated genes, whereas variants of uncertain significance were not classified as genotype positive. There is still a possibility that other pathogenic variants exist, and this classification may be updated in the near future.

## Conclusions

The deep learning model had modest accuracy in predicting genotype positivity using echocardiographic videos, and the Madrid genotype score improved the accuracy when combined with the deep learning model. Echocardiographic videos have the potential to serve as an adjunctive tool to clinical scores for predicting genotype positivity.

## Supplementary Material

ztag068_Supplementary_Data

## Data Availability

The echocardiographic video dataset is not publicly available due to Institutional Review Board stipulations.

## References

[1] Arbelo E, Protonotarios A, Gimeno JR, Arbustini E, Barriales-Villa R, Basso C, et al 2023 ESC Guidelines for the management of cardiomyopathies. Eur Heart J 2023;44:3503–3626. 37622657 10.1093/eurheartj/ehad194

[2] Tayal U, Prasad S, Cook SA. Genetics and genomics of dilated cardiomyopathy and systolic heart failure. Genome Med 2017;9:20. 28228157 10.1186/s13073-017-0410-8 PMC5322656

[3] Tayal U, Ware JS, Lakdawala NK, Heymans S, Prasad SK. Understanding the genetics of adult-onset dilated cardiomyopathy: what a clinician needs to know. Eur Heart J 2021;42:2384–2396. 34153989 10.1093/eurheartj/ehab286 PMC8216730

[4] Escobar-Lopez L, Ochoa JP, Mirelis JG, Espinosa MÁ, Navarro M, Gallego-Delgado M, et al Association of genetic variants with outcomes in patients with nonischemic dilated cardiomyopathy. J Am Coll Cardiol 2021;78:1682–1699. 34674813 10.1016/j.jacc.2021.08.039

[5] Gigli M, Merlo M, Graw SL, Barbati G, Rowland TJ, Slavov DB, et al Genetic risk of arrhythmic phenotypes in patients with dilated cardiomyopathy. J Am Coll Cardiol 2019;74:1480–1490. 31514951 10.1016/j.jacc.2019.06.072 PMC6750731

[6] Pasotti M, Klersy C, Pilotto A, Marziliano N, Rapezzi C, Serio A, et al Long-term outcome and risk stratification in dilated cardiolaminopathies. J Am Coll Cardiol 2008;52:1250–1260. 18926329 10.1016/j.jacc.2008.06.044

[7] Longoni M, Bhasin K, Ward A, Lee D, Nisson M, Bhatt S, et al Real-world utilization of guideline-directed genetic testing in inherited cardiovascular diseases. Front Cardiovasc Med 2023;10:1272433. 37915745 10.3389/fcvm.2023.1272433 PMC10616303

[8] Elias P, Jain S, Poterucha T, Randazzo M, Lopez Jimenez F, Khera R, et al Artificial intelligence for cardiovascular care—part 1: advances: JACC review topic of the week. J Am Coll Cardiol 2024;83:2472–2486. 38593946 10.1016/j.jacc.2024.03.400

[9] Ouyang D, He B, Ghorbani A, Yuan N, Ebinger J, Langlotz CP, et al Video-based AI for beat-to-beat assessment of cardiac function. Nature 2020;580:252–256. 32269341 10.1038/s41586-020-2145-8 PMC8979576

[10] Yuan N, Stein NR, Duffy G, Sandhu RK, Chugh SS, Chen P-S, et al Deep learning evaluation of echocardiograms to identify occult atrial fibrillation. NPJ Digit Med 2024;7:96. 38615104 10.1038/s41746-024-01090-z PMC11016113

[11] Katsushika S, Kodera S, Nakamoto M, Ninomiya K, Kakuda N, Shinohara H, et al Deep learning algorithm to detect cardiac sarcoidosis from echocardiographic movies. Circ J 2021;86:87–95. 34176867 10.1253/circj.CJ-21-0265

[12] Richardson P, McKenna W, Bristow M, Maisch B, Mautner B, O’Connell J, et al Report of the 1995 World Health Organization/International Society and Federation of Cardiology Task Force on the Definition and Classification of cardiomyopathies. Circulation 1996;93:841–842. 8598070 10.1161/01.cir.93.5.841

[13] Bozkurt B, Colvin M, Cook J, Cooper LT, Deswal A, Fonarow GC, et al Current diagnostic and treatment strategies for specific dilated cardiomyopathies: a scientific statement from the American Heart Association. Circulation 2016;134:e579–e646. 27832612 10.1161/CIR.0000000000000455

[14] Inoue S, Ko T, Shindo A, Nomura S, Yamada T, Jimba T, et al Association between clonal hematopoiesis and left ventricular reverse remodeling in nonischemic dilated cardiomyopathy. JACC Basic Transl Sci 2024;9:956–967. 39297129 10.1016/j.jacbts.2024.04.010 PMC11405799

[15] Richards S, Aziz N, Bale S, Bick D, Das S, Gastier-Foster J, et al Standards and guidelines for the interpretation of sequence variants: a joint consensus recommendation of the American College of Medical Genetics and Genomics and the Association for Molecular Pathology. Genet Med 2015;17:405–424. 25741868 10.1038/gim.2015.30 PMC4544753

[16] Escobar-Lopez L, Ochoa JP, Royuela A, Verdonschot JAJ, Dal Ferro M, Espinosa MA, et al Clinical risk score to predict pathogenic genotypes in patients with dilated cardiomyopathy. J Am Coll Cardiol 2022;80:1115–1126. 36109106 10.1016/j.jacc.2022.06.040 PMC10804447

[17] Paszke A, Gross S, Massa F, Lerer A, Bradbury J, Chanan G, et al PyTorch: An Imperative Style, High-Performance Deep Learning Library. arXiv, arXiv:1912.01703.

[18] Ntusi NAB, Sliwa K. Impact of racial and ethnic disparities on patients with dilated cardiomyopathy: JACC focus seminar 7/9. J Am Coll Cardiol 2021;78:2580–2588. 34887144 10.1016/j.jacc.2021.10.021

[19] Myers VD, Gerhard GS, McNamara DM, Tomar D, Madesh M, Kaniper S, et al Association of variants in BAG3 with cardiomyopathy outcomes in African American individuals. JAMA Cardiol 2018;3:929–938. 30140897 10.1001/jamacardio.2018.2541 PMC6233818

[20] Morales A, Moretz C, Ren S, Smith E, Callis TE, Hall T, et al Real-world genetic testing utilization among patients with cardiomyopathy. Circ Genom Precis Med 2024;17:e004028. 38088168 10.1161/CIRCGEN.122.004028

[21] Zeljkovic I, Gauthey A, Manninger M, Malaczynska-Rajpold K, Tfelt-Hansen J, Crotti L, et al Genetic testing for inherited arrhythmia syndromes and cardiomyopathies: results of the European Heart Rhythm Association survey. Europace 2024;26:euae216. 39148456 10.1093/europace/euae216 PMC11411205

[22] Heidenreich PA, Bozkurt B, Aguilar D, Allen LA, Byun JJ, Colvin MM, et al 2022 AHA/ACC/HFSA guideline for the management of heart failure: a report of the American College of Cardiology/American Heart Association Joint Committee on Clinical Practice Guidelines. Circulation 2022;145:e895–e1032. 35363499 10.1161/CIR.0000000000001063

[23] Liu Z, Duan T, Zhang Y, Weng S, Xu H, Ren Y, et al Radiogenomics: a key component of precision cancer medicine. Br J Cancer 2023;129:741–753. 37414827 10.1038/s41416-023-02317-8 PMC10449908

[24] Guo Y, Li T, Gong B, Hu Y, Wang S, Yang L, et al From images to genes: radiogenomics based on artificial intelligence to achieve non-invasive precision medicine in cancer patients. Adv Sci (Weinh) 2025;12:e2408069. 39535476 10.1002/advs.202408069 PMC11727298

