# Supplementary material for: Deep learning model for genotype prediction from echocardiographic videos in non-ischaemic dilated cardiomyopathy
Source: Eur Heart J Digit Health. 2026 May 5;7(5):ztag068. doi: 10.1093/ehjdh/ztag068 (PMC13188222; doi:10.1093/ehjdh/ztag068)
Supplement: ztag068_Supplementary_Data [file ztag068_supplementary_data.docx]

**SUPPLEMENTARY MATERIALS**

**Deep Learning Model for Genotype Prediction from Echocardiographic Videos**

**in Non-Ischemic Dilated Cardiomyopathy**

Yuko Kiyohara, MD, Seito Fukagawa, MD, Seitaro Nomura, MD, PhD, Satoshi Kodera, MD, PhD, Koki Nakanishi, MD, PhD, Takashi Hiruma, MD, Ryo Abe, MD, Shunsuke Inoue, MD, PhD, Junichi Ishida, MD, PhD, Eisuke Amiya, MD, PhD, Masaru Hatano, MD, PhD, Hiroyuki Morita, MD, PhD, Norihiko Takeda, MD, PhD, Issei Komuro, MD, PhD

Table of Contents

[Supplementary Table 1. The list of 70 genes in the targeted sequencing or whole-exome sequencing. 2](#_Toc222847897)

[Supplementary Table 2. Summary of germline variants identified in cardiomyopathy-related genes. 3](#_Toc222847898)

[Supplementary Table 3. Comparison of echocardiographic features between cases predicted as genotype-positive and those predicted as genotype-negative by the deep learning model. 6](#_Toc222847899)

[Supplementary Figure 1. The summary of the whole process of the development of the deep learning model. 7](#_Toc222847900)

[Supplementary Figure 2. The ROC curves of the deep learning models for subtype variants. 8](#_Toc222847901)

# **Supplementary Table 1. The list of 70 genes in the targeted sequencing or whole-exome sequencing.**

| *ABCC9* | *ACTC1* | *ACTN2* | *AKAP9* | *ANK2* | *ANKRD1* | *BAG3* |
| --- | --- | --- | --- | --- | --- | --- |
| *BAG5* | *CACNB2* | *CALR3* | *CASQ2* | *CAV3* | *CRYAB* | *CSRP3* |
| *DES* | *DMD* | *DSC2* | *DSG2* | *DSP* | *EMD* | *EYA4* |
| *FLNC* | *FXN* | *GLA* | *GPD1L* | *ILK* | *JPH2* | *JUP* |
| *KCNE1* | *KCNE2* | *KCNE3* | *KCNH2* | *KCNJ2* | *KCNQ1* | *LAMP2* |
| *LDB3* | *LMNA* | *MYBPC3* | *MYH6* | *MYH7* | *MYL2* | *MYL3* |
| *MYOZ2* | *MYPN* | *NEBL* | *NEXN* | *NKX2-5* | *PDLIM3* | *PKP2* |
| *PLN* | *PRKAG2* | *RAF1* | *RBM20* | *RYR2* | *SCN1B* | *SCN3B* |
| *SCN4B* | *SCN5A* | *SGCD* | *TAZ* | *TCAP* | *TGFB3* | *TMEM43* |
| *TNNC1* | *TNNI3* | *TNNT2* | *TPM1* | *TTN* | *TXNRD2* | *VCL* |

# **Supplementary Table 2. Summary of germline variants identified in cardiomyopathy-related genes.**

| **Chr** | **Start** | **Gene** | **NM** | **Codon** | **Amino acid** | **ACMG** |
| --- | --- | --- | --- | --- | --- | --- |
| chr15 | 34792531 | *ACTC1* | NM_005159 | c.G493A | p.V165I | LP |
| chr1 | 236755157 | *ACTN2* | NM_001103 | c.G2113A | p.A705T | LP |
| chr10 | 119672527 | *BAG3* | NM_001001430 | c.780_794del | p.R261_P265del | LP |
| chr10 | 119676605 | *BAG3* | NM_004281 | c.1051_1058del | p.V351Efs*7 | LP |
| chr10 | 119676917 | *BAG3* | NM_004281 | c.G1363A | p.E455K | LP |
| chr10 | 119677261 | *BAG3* | NM_004281 | c.1707delT | p.G570Vfs*25 | P |
| chr2 | 219418830 | *DES* | NM_001927 | c.T368A | p.I123N | LP |
| chr2 | 219421550 | *DES* | NM_001927 | c.G1234A | p.E412K | LP |
| chr18 | 31520941 | *DSG2* | NM_001943 | c.C355T | p.R119X | P |
| chr18 | 31536395 | *DSG2* | NM_001943 | c.1617_1644del | p.M540Kfs*23 | LP |
| chr6 | 7565389 | *DSP* | NM_004415 | c.C808T | p.R270X | P |
| chr6 | 7575400 | *DSP* | NM_004415 | c.2542_2543insTTTA | p.D850Lfs*2 | LP |
| chr6 | 7575446 | *DSP* | NM_004415 | c.T2588C | p.L863P | LP |
| chr6 | 7579897 | *DSP* | NM_004415 | c.3708dupA | p.N1237Kfs*4 | LP |
| chr6 | 7583113 | *DSP* | NM_004415 | c.C5851T | p.R1951X | P |
| chrX | 154381015 | *EMD* | NM_000117 | c.583delT | p.S195Hfs*42 | LP |
| chrX | 154381109 | *EMD* | NM_000117 | c.G677A | p.W226X | LP |
| chr7 | 128837721 | *FLNC* | NM_001458 | c.935_936insCA | p.Y313Tfs*39 | LP |
| chr7 | 128838780 | *FLNC* | NM_001458 | c.1388_1391del | p.F464Lfs*27 | LP |
| chr7 | 128838783 | *FLNC* | NM_001458 | c.1391_1392insAGGGAAGG | p.F464Lfs*31 | LP |
| chr7 | 128840135 | *FLNC* | NM_001458 | c.1524_1525insCTCAAGGTCA | p.V513Qfs*53 | LP |
| chr7 | 128853975 | *FLNC* | NM_001458 | c.6486delA | p.N2163Tfs*12 | LP |
| chr7 | 128857196 | *FLNC* | NM_001458 | c.7640_7644del | p.K2547Tfs*9 | LP |
| chr1 | 156115015 | *LMNA* | NM_170707 | c.G97T | p.E33X | LP |
| chr1 | 156115072 | *LMNA* | NM_170707 | c.C154G | p.L52V | LP |
| chr1 | 156115096 | *LMNA* | NM_170707 | c.C178A | p.R60S | LP |
| chr1 | 156115199 | *LMNA* | NM_170707 | c.C281G | p.S94X | LP |
| chr1 | 156134457 | *LMNA* | NM_170707 | c.C568T | p.R190W | P |
| chr1 | 156134458 | *LMNA* | NM_170707 | c.G569A | p.R190Q | P |
| chr1 | 156134795 | *LMNA* | NM_170707 | NA | NA | P |
| chr1 | 156134911 | *LMNA* | NM_170707 | c.G746A | p.R249Q | LP |
| chr1 | 156134937 | *LMNA* | NM_170707 | c.C772T | p.Q258X | LP |
| chr1 | 156135280 | *LMNA* | NM_170707 | c.904_905del | p.S303Cfs*27 | P |
| chr1 | 156135293 | *LMNA* | NM_170707 | c.T917C | p.L306P | LP |
| chr1 | 156135900 | *LMNA* | NM_170707 | c.937-1G>A | NA | P |
| chr1 | 156135967 | *LMNA* | NM_170707 | c.C1003T | p.R335W | LP |
| chr1 | 156136022 | *LMNA* | NM_170707 | c.A1058G | p.Q353R | P |
| chr1 | 156136952 | *LMNA* | NM_170707 | c.G1412A | p.R471H | LP |
| chr1 | 156137138 | *LMNA* | NM_170707 | c.1514delC | p.H506Tfs*42 | P |
| chr1 | 156137666 | *LMNA* | NM_170707 | c.C1621T | p.R541C | LP |
| chr1 | 156137667 | *LMNA* | NM_170707 | c.G1622A | p.R541H | P |
| chr14 | 23425004 | *MYH7* | NM_000257 | c.A2444T | p.Q815L | LP |
| chr14 | 23427257 | *MYH7* | NM_000257 | c.G1939A | p.V647M | LP |
| chr14 | 23427857 | *MYH7* | NM_000257 | c.T1616C | p.M539T | LP |
| chr14 | 23431798 | *MYH7* | NM_000257 | c.T602C | p.I201T | LP |
| chr1 | 77935997 | *NEXN* | NM_144573 | c.G1426C | p.A476P | LP |
| chr10 | 110644617 | *RBM20* | NM_001134363 | c.C163T | p.Q55X | LP |
| chr10 | 110812297 | *RBM20* | NM_001134363 | c.C1900T | p.R634W | LP |
| chr10 | 110821365 | *RBM20* | NM_001134363 | c.2746_2748del | p.E918del | LP |
| chr3 | 38613781 | *SCN5A* | NM_198056 | c.G665A | p.R222Q | P |
| chr3 | 52451512 | *TNNC1* | NM_003280 | c.C333G | p.Y111X | LP |
| chr3 | 52452169 | *TNNC1* | NM_003280 | c.A139C | p.M47L | LP |
| chr19 | 55156626 | *TNNI3* | NM_000363 | c.G127A | p.A43T | LP |
| chr1 | 201359242 | *TNNT2* | NM_001001430 | c.G835A | p.G279R | LP |
| chr1 | 201361971 | *TNNT2* | NM_001001430 | c.629_631del | p.K210del | P |
| chr1 | 201361989 | *TNNT2* | NM_001001430 | c.C613T | p.R205W | LP |
| chr1 | 201364335 | *TNNT2* | NM_001001430 | c.G422A | p.R141Q | LP |
| chr1 | 201365244 | *TNNT2* | NM_001001430 | c.T328A | p.F110I | LP |
| chr1 | 201365298 | *TNNT2* | NM_001001430 | c.C274T | p.R92W | LP |
| chr15 | 63057015 | *TPM1* | NM_001018005 | c.C271T | p.R91C | LP |
| chr15 | 63062263 | *TPM1* | NM_001018005 | c.G688A | p.D230N | P |
| chr2 | 178528593 | *TTN* | NM_133378 | c.99453_99454insA | p.L33152Tfs*5 | LP |
| chr2 | 178532100 | *TTN* | NM_133378 | c.C96811T | p.R32271X | P |
| chr2 | 178532844 | *TTN* | NM_133378 | c.C96067T | p.R32023X | P |
| chr2 | 178543878 | *TTN* | NM_133378 | c.88562delA | p.N29521Tfs*24 | LP |
| chr2 | 178546699 | *TTN* | NM_133378 | c.G87025T | p.G29009X | P |
| chr2 | 178550223 | *TTN* | NM_133378 | c.83899_83911del | p.G27967Sfs*20 | P |
| chr2 | 178552086 | *TTN* | NM_133378 | c.83107_83110del | p.N27703Gfs*18 | LP |
| chr2 | 178552162 | *TTN* | NM_133378 | c.G83034A | p.W27678X | LP |
| chr2 | 178554684 | *TTN* | NM_133378 | c.80958_80959del | p.W26987Afs*5 | LP |
| chr2 | 178560006 | *TTN* | NM_133378 | c.78409_78422del | p.L26137Ifs*11 | P |
| chr2 | 178560283 | *TTN* | NM_133378 | c.G78145T | p.E26049X | LP |
| chr2 | 178563607 | *TTN* | NM_133378 | c.C74821T | p.R24941X | P |
| chr2 | 178566283 | *TTN* | NM_133378 | c.A72145T | p.K24049X | LP |
| chr2 | 178566331 | *TTN* | NM_133378 | c.G72097T | p.G24033X | P |
| chr2 | 178566463 | *TTN* | NM_133378 | c.C71965T | p.Q23989X | LP |
| chr2 | 178566556 | *TTN* | NM_133378 | c.71872delA | p.I23958Sfs*11 | LP |
| chr2 | 178570991 | *TTN* | NM_133378 | c.67434_67437del | p.K22478Nfs*8 | P |
| chr2 | 178575154 | *TTN* | NM_133378 | c.C63274T | p.R21092X | P |
| chr2 | 178575249 | *TTN* | NM_133378 | c.63158_63179del | p.P21053Qfs*38 | LP |
| chr2 | 178577085 | *TTN* | NM_133378 | c.C61546T | p.R20516X | P |
| chr2 | 178578066 | *TTN* | NM_133378 | c.C60745T | p.R20249X | P |
| chr2 | 178579623 | *TTN* | NM_133378 | c.59870delC | p.A19957Vfs*28 | LP |
| chr2 | 178590395 | *TTN* | NM_133378 | c.A53626T | p.R17876X | LP |
| chr2 | 178597751 | *TTN* | NM_133378 | c.C49627T | p.R16543X | P |
| chr2 | 178599221 | *TTN* | NM_133378 | c.C48868T | p.R16290X | P |
| chr2 | 178609462 | *TTN* | NM_133378 | c.44144delA | p.K14715Rfs*36 | LP |
| chr2 | 178609842 | *TTN* | NM_133378 | c.G43877A | p.W14626X | P |
| chr2 | 178610323 | *TTN* | NM_133378 | c.G43499A | p.W14500X | LP |
| chr2 | 178612442 | *TTN* | NM_133378 | c.C42379T | p.R14127X | P |
| chr2 | 178634519 | *TTN* | NM_133378 | c.G34558T | p.G11520X | P |
| chr2 | 178634533 | *TTN* | NM_133378 | c.34522_34544del | p.V11508Yfs*7 | LP |
| chr2 | 178635576 | *TTN* | NM_133378 | c.34044delA | p.E11348Dfs*9 | P |
| chr2 | 178639788 | *TTN* | NM_133378 | c.33082_33083insGAGGTGGTTTGATTGTTTTCACTTCTGTAGAGAG | p.E11028Gfs*24 | LP |
| chr2 | 178759074 | *TTN* | NM_133378 | c.G10213T | p.E3405X | LP |
| chr2 | 178768814 | *TTN* | NM_133378 | c.9022delA | p.I3008Sfs*9 | LP |

ACMG: American College of Medical Genetics and Genomics, NM: National Center for Biotechnology Information RefSeq mRNA, LP: likely pathogenic, P: pathogenic.

# **Supplementary Table 3. Comparison of echocardiographic features between cases predicted as genotype-positive and those predicted as genotype-negative by the deep learning model.**

|  | **Predicted Genotype-positive Group**  (n = 97) | **Predicted Genotype-negative**  **Group**  (n = 136) | ***P* value** |
| --- | --- | --- | --- |
| **LV end-diastolic diameter (mm)** | 64 ± 11 | 68 ± 11 | 0.022* |
| **LV end-systolic diameter (mm)** | 56 ± 13 | 60 ± 13 | 0.023* |
| **LVEF (%)** | 27 ± 14 | 26 ± 13 | 0.633 |
| **RVSP (mmHg)** | 30 ± 15 | 33 ± 13 | 0.138 |
| **TAPSE (mm)** | 16 ± 5 | 17 ± 7 | 0.279 |

LV: left ventricle, LVEF: left ventricular ejection fraction, RVSP: right ventricular systolic pressure, TAPSE: tricuspid annular plane systolic excursion.

# **Supplementary Figure 1. The summary of the whole process of the development of the deep learning model.**

**
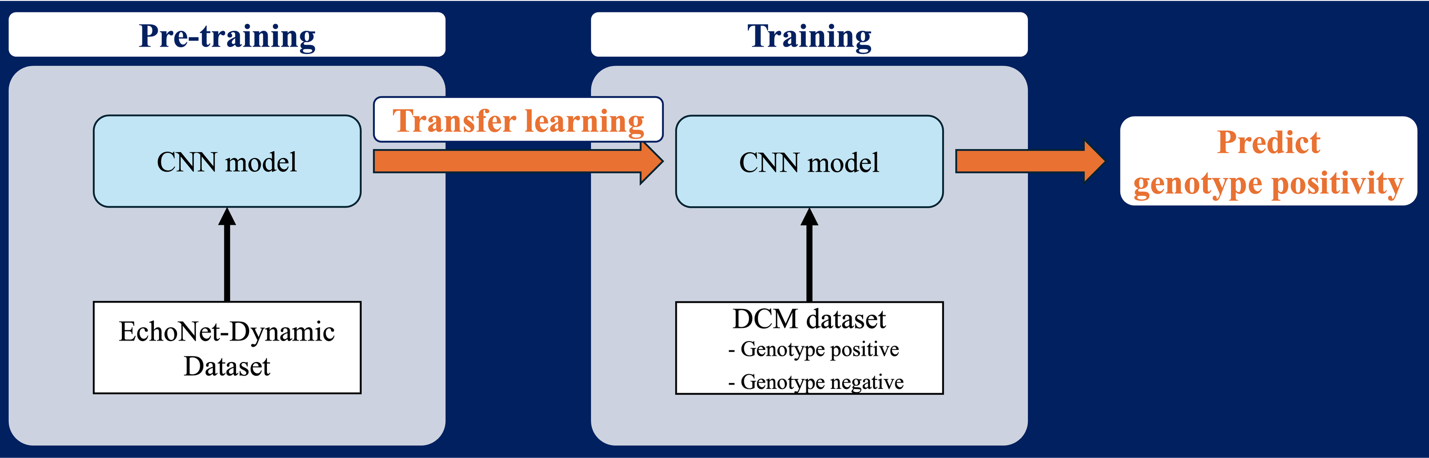
**

CNN: convolutional neural network, DCM: dilated cardiomyopathy.

# **Supplementary Figure 2. The ROC curves of the deep learning models for subtype variants.**

(A) The deep learning model to predict *LMNA* pathogenic variants. The AUC was calculated as 0.49. (B) The deep learning model to predict sarcomere gene variants. The AUC was calculated as 0.59.

(A) The deep learning model to predict *LMNA* pathogenic variants


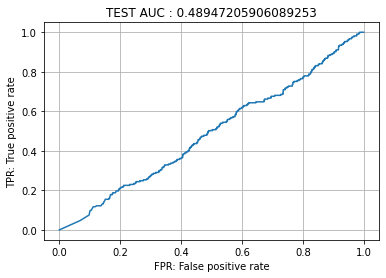


(B) The deep learning model to predict sarcomere gene variants


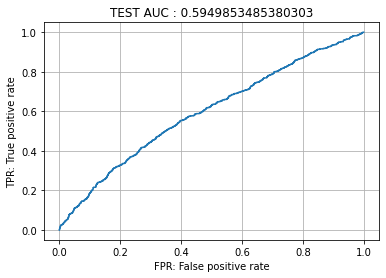


AUC: area under the curve, ROC: receiver operating characteristic.
